# Elevated Levels of Serum IL-12 and IL-18 are Associated with Lower Frequencies of CD4^+^CD25^high^FOXP3^+^ Regulatory T cells in Young Patients with Type 1 Diabetes

**DOI:** 10.1007/s10753-014-9878-1

**Published:** 2014-03-28

**Authors:** Monika Ryba-Stanisławowska, Karolina Rybarczyk-Kapturska, Małgorzata Myśliwiec, Jolanta Myśliwska

**Affiliations:** 1 Department of Immunology, Medical University of Gdańsk, Dębinki 1, 80-210 Gdańsk, Poland; 2 Clinic of Pediatrics, Department of Diabetology and Endocrinology, Medical University of Gdańsk, 80-210 Gdańsk, Poland

**Keywords:** diabetes type 1, IL-18, IL-12, tregs, inflammation

## Abstract

Type 1 diabetes is thought to involve chronic inflammation, which is manifested by the activation and expression of different inflammatory mediators. IL-12 and IL-18 are two cytokines that have been shown to exert strong proinflammatory activity and have been implicated in the pathogenesis of type 1 diabetes in mice and humans. The overproduction of proinflammatory mediators is controlled by specialized T cell subset, namely regulatory T cells that express FOXP3 transcription factor. Since IL-12 and IL-18 mediate inflammatory response and Tregs exhibit anti-inflammatory potential, we aimed to examine their reciprocal relationship in patients with type 1 diabetes. The study group consisted of 47 children diagnosed with type 1 diabetes and 28 healthy individuals. Serum levels of IL-12 and IL-18 were measured by ELISA, and the peripheral blood CD4^+^CD25^high^ FOXP3^+^ regulatory T cell frequencies were analyzed by flow cytometry. Patients with type 1 diabetes had a decreased percentage of circulating CD4^+^CD25^high^FOXP3^+^ Tregs in comparison to their healthy counterparts. In addition, they produced more IL-12 and IL-18 than children from the control group. Concentrations of these cytokines positively correlated with one another, as well as with CRP and HbA1c. Moreover, the negative association between IL-12, IL-18, CRP serum levels, and the frequency of regulatory CD4^+^CD25^high^FOXP3^+^ Tregs was observed. IL-12 and IL-18 may have direct or indirect impact on regulatory T cell subset, which may contribute to their reduced frequency in peripheral blood of patients with type 1 diabetes mellitus.

## INTRODUCTION

Type 1 diabetes mellitus (DM1) is thought to involve chronic inflammation, which is manifested by the activation and expression of different inflammatory mediators [1]. As a result, various diabetic complications develop leading to increased mortality and morbidity.

IL-12 is a proinflammatory cytokine produced by antigen presenting cells in response to PAMPs (pathogen-associated molecular patterns) and DAMPs (danger-associated molecular patterns). It induces the polarization of the immune response towards Th1 profile, which protects against intracellular pathogens [2, 3]. IL-12 has been implicated in the pathogenesis of type 1 diabetes in the NOD (non-obese diabetic) mouse [4]. Alleva *et al.* showed that macrophages from NOD mice produced more IL-12 than NOR (non-obese resistant) mice macrophages [5]. A link between IL-12 and type 1 diabetes was also suggested in humans. Glucose-stimulated PBMCs (peripheral blood mononuclear cells) from healthy subjects produced more IL-12 than resting, unstimulated cells [6]. What’s more, the production of IL-12 did not change even after insulin treatment [6]. Similar effect was seen in patients with type 2 diabetes. The LPS-stimulated PBMCs under glucose treatment produced elevated level of IL-12 [7]. Furthermore, it was shown that patients with longstanding DM1 show increased levels of IL-12 in both serum and aqueous humor [8]. The latter may provide an evidence for the involvement of IL-12 in pathogenesis of late diabetic microvascular complications.

IL-18 belongs to the IL-1 superfamily of cytokines and similarly to IL-1 it is synthesized as inactive precursor and is secreted when appropriate cleaving enzymes are present [9]. In synergy with IL-12, IL-18 activates polarization of Th1 cells, augments activity of NK cells, and induces IFN-γ production [10]. IL-18 was shown to play a role in pathogenesis of inflammatory diseases such as thyroid destruction in Hashimoto’s thyroiditis [11], rheumatoid arthritis [12, 13], allergy, asthma [14, 15], and Crohn’s disease [16]. Some authors also linked IL-18 or its receptor polymorphism with type 1 diabetes [17–21]. Furthermore, studies done by others showed that IL-18 serum concentrations are elevated in patients with type 2 diabetes [22, 23] and/or diabetic nephropathy [24, 25].

Regulatory T cells (Tregs) play a crucial role in the maintenance of immune homeostasis in controlling autoimmunity and inflammation. They are responsible for suppressing the excessive ability of different cells to proliferate and/or produce proinflammatory cytokines [26, 27]. They are characterized by the coexpression of CD4, CD25, and a transcription factor FOXP3, thus the CD4^+^CD25^high^FOXP3^+^ is the most widely accepted phenotype of Tregs [28, 29]. Defects in Tregs have been reported by us and others in several autoimmune/inflammatory diseases such as multiple sclerosis, rheumatoid arthritis, systemic lupus erythematosus, or type 1 diabetes [27, 30–33]. Moreover, it was shown that depletion of Tregs deteriorated nephropathy in non-insulin-dependent diabetic mice, while adoptive transfer of Tregs exerted protective effect on kidneys [34]. Immune response studies in animal models of kidney injury also suggested the protective role for the CD4^+^FOXP3^+^ regulatory T cell subset [35–37].

Since IL-12 and IL-18 mediate inflammatory response and Tregs exhibit anti-inflammatory potential, we aimed to examine the relation between them in patients with type 1 diabetes. It is particularly important because the disease can progress into clinically manifested vascular complications such as retinopathy or nephropathy.

## MATERIALS AND METHODS

### Subjects

The study group consisted of 47 young patients (mean age; 14.25 ± 3.5 years) diagnosed with type 1 diabetes that were recruited from Clinic of Pediatrics, Department of Diabetology and Endocrinology Medical University of Gdańsk. Type 1 diabetes was defined according to the criteria of the American Diabetes Association. Patients with microvascular complications, as well as those with coexisting autoimmune, chronic, and acute inflammatory diseases were excluded from the study. The mean duration of the disease was 7.39 ± 3.8 years. In all examined patients, the C-peptide levels were below 0.5 ng/ml. All patients were treated with humanized insulin at doses of 0.87 ± 0.2 mg/kg. At the time of sampling, a blood glucose level along with biochemical measurement of renal function, lipid status, C-reactive protein (CRP), and glycosylated hemoglobin (HbA1c) were monitored.

The control group consisted of 28 age and sex-matched individuals recruited during control visits in outpatient clinic. No signs of autoimmune, chronic, inflammatory, and neoplastic disease at the time of sampling and no evidence of DM1 in their families were disclosed as confirmed by medical records, laboratory examination, and laboratory tests.

All subjects gave informed consent and the study followed the principles of the Declaration of Helsinki and was approved by The Ethics Committee of The Medical University of Gdańsk.

### Isolation and Flow Cytometric Analysis of Peripheral Blood CD4^+^CD25^high^ FOXP3^+^ Regulatory T cells

Heparinised venous blood samples were collected and used to isolate PBMCs (peripheral blood mononuclear cells) by density gradient preparation over Ficoll-Uropoline. 1 × 10^6^ freshly isolated PBMCs were destined for flow cytometric staining. Cells were stained with anti-CD4 (IgG1,κ mouse Pe/Cy5, Clone RPA-T4, BioLegend, USA) and anti-CD25 (IgG1, ĸ mouse PE, Clone BC96, BioLegend, USA) antibodies and incubated for 30 min in the dark, fixed, and stained for intracellular expression of FOXP3 (IgG1,κ mouse Alexa-Fluor 488, Clone 206D, BioLegend, USA). Measurements were performed on the LSRII flow cytometer (BD Biosciences). Dead cells were excluded by forward and side scatter. Positive signal for each staining was established using appropriate isotype control. Data were analyzed by FACSDiva 6.0 Software (Becton Dickinson, USA).

### Determination of Serum IL-12 and IL-18 Levels

Serum levels of IL-12 and IL-18 were measured by ELISA method (Quantikine R&D Systems, Minneapolis, Minn., USA and Quantitative test, MBL International, USA, respectively). According to the manufacturer protocol, minimum detectable concentrations were determined by the manufacturer as 0.1 pg/ml for IL-12 and 12.5 pg/ml for IL-18.

Intra-assay coefficient of variation ranged between 2.5–4.9 % (IL-12) and 5.03–9.92 % (IL-18). The inter-assay coefficient of variation was 7.6–12.6 % (IL-12) and 6.25–10.07 % (IL-18). The results were read on the automated plate reader (Multiscan MCC/340, Labsystems, Helsinki, Finland).

### Statistical Analysis

The results were analyzed using the Statistica, ver. 10.0 (StatSoft Inc, USA). For comparison of the skew-distributed variables, non-parametric Mann-Whitney U test was applied. Spearman’s correlations were used to compare the associations between analyzed parameters. The level of significance was set at *p* ≤ 0.05.

## RESULTS

### Clinical Characteristics of the Study Groups

The basic characteristic of children enrolled in the study is presented in Table 1. No significant difference was detected between diabetic and control group by means of age, gender, and BMI. Patients with type 1 diabetes had significantly higher levels of HbA1c, as well as CRP in comparison to the age and sex-matched healthy individuals from the control group.

**Table 1:** General Clinical Characteristics of Children with Type 1 Diabetes and Healthy Individuals

Group	Age (years)	Gender (F/M)	Disease duration (years)	BMI (kg/m^2^)	HbA_1_c (%)	Albumin excretion rate(mg/24 h)	CRP mg/l
DM1 (*n* = 47)	14.25 ± 3.58	26/21	7.39 ± 3.8	18.4 ± 3.3	8.7 ± 2.26	17.7 ± 6.9	2.41 ± 1.89
healthy (*n* = 28)	15.6 ± 1.7	16/14	–	17.8 ± 3.2	5.2 ± 0.1	–	0.7±0.15
p*	0.7	0.8		0.2	0.001		0.0002

Data are shown as mean ± SD

*The significance between DM1 patients and healthy subjects

### Serum Concentrations of IL-12 and IL-18 in Patients with Type 1 Diabetes

**Fig. 1:**
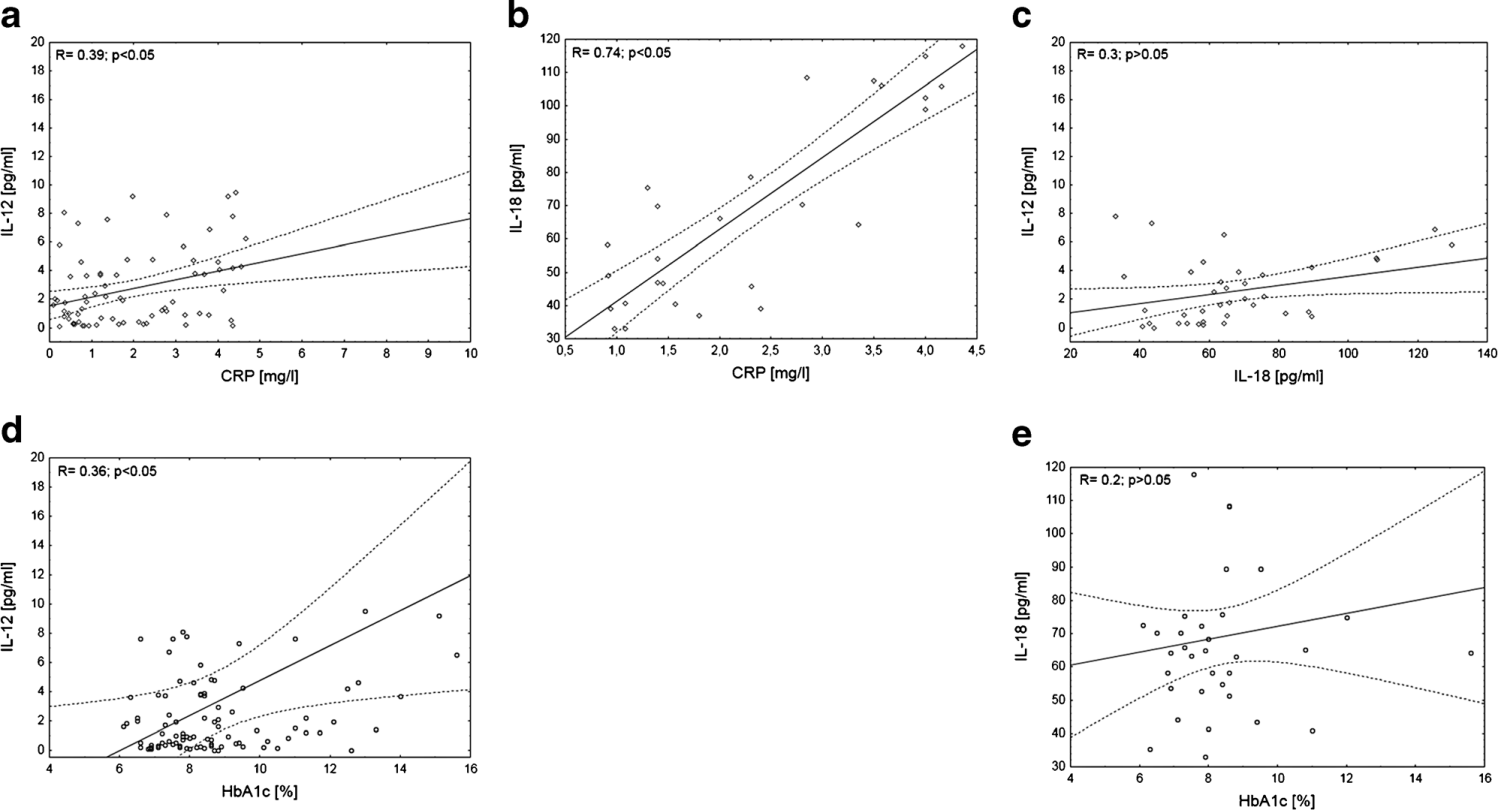
Relationship between serum level of IL-12, IL-18 CRP, and HbA1c in patients with type 1 diabetes. The levels of IL-12, IL-18 CRP, and HbA1c were measured in the blood of DM1 children and correlated with each other. The Spearman test was used to calculate the strength of correlation. **a** The correlation between IL-12 and CRP serum level in DM1 subjects (*R* = 0.39; *p* < 0.05); **b** The correlation between IL-18 and CRP serum level in DM1 subjects (*R* = 0.74; *p* < 0.05); **c** The correlation between IL-12 and IL-18 serum level in DM1 subjects (*R* = 0.3; *p* > 0.05). **d** The correlation between IL-12 and HbA1c in DM1 subjects (*R* = 0.36; *p* < 0.05); **e** The correlation between IL-18 and HbA1c level in DM1 subjects (*R* = 0.2; *p* > 0.05).

Patients with type 1 diabetes showed statistically higher serum levels of IL-12 and IL-18 than children from the control group (Table 2). Concentrations of these cytokines positively correlated with CRP level (Fig. 1a, b) as well as demonstrated a positive correlation with one another (Fig. 1c). As to the association of analyzed cytokines with HbA1c, we found correlation only in case of IL-12 (Fig. 1d). We could not found significant correlation between serum IL-18 and HbA1c (Fig. 1e).

**Table 2:** Serum Level of IL-12 and IL-18

	DM1 patients	Control group	*p*
IL-12 (pg/ml)	1.2 (0.2/3.8)	0.1 (0/0.4)	0.00001
IL-18 (pg/ml)	64.8 (42.9/108.2)	44.4 (43.5/66.1)	0.006

The results are shown as median and 10/90 percentile. All the differences were calculated by the Mann-Whitney U test

### Peripheral Blood CD4^+^CD25^high^FOXP3^+^ Regulatory T Cell Counts in Patients with Type 1 Diabetes

**Fig. 2:**
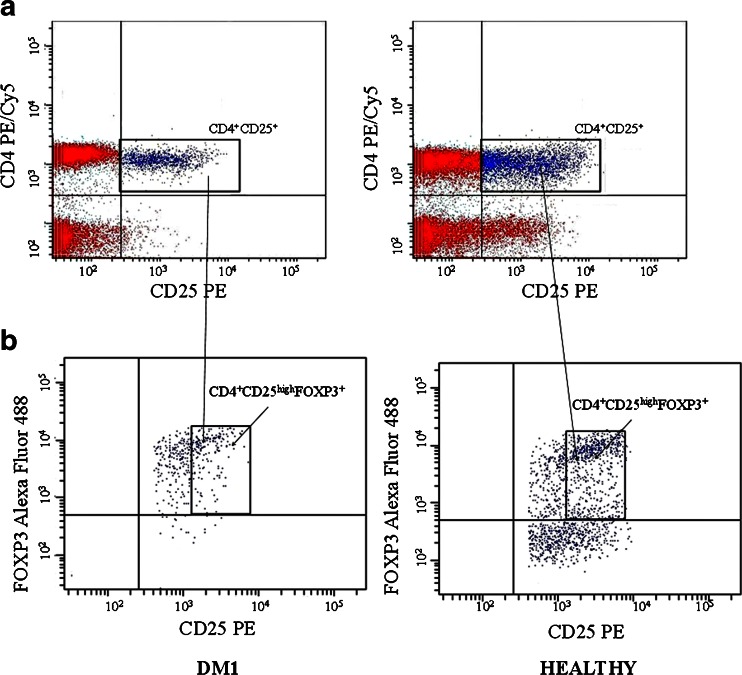
Representative staining of circulating CD4^+^CD25^high^FOXP3^+^ T cells in patient with type 1 diabetes and healthy individual. Fresh, resting PBMCs from diabetic type 1 patients and healthy individuals were stained with antibodies against CD4, CD25, and FOXP3 molecules and analyzed using flow cytometry. The gate was set on CD4^+^ CD25^+^ lymphocytes **a** Based on the CD4^+^CD25^+^ gate, cells were further gated based on CD25 and FOXP3 expression and the frequency of CD4^+^CD25^high^FOXP3^+^ cells was determined **b**.

**Fig. 3:**
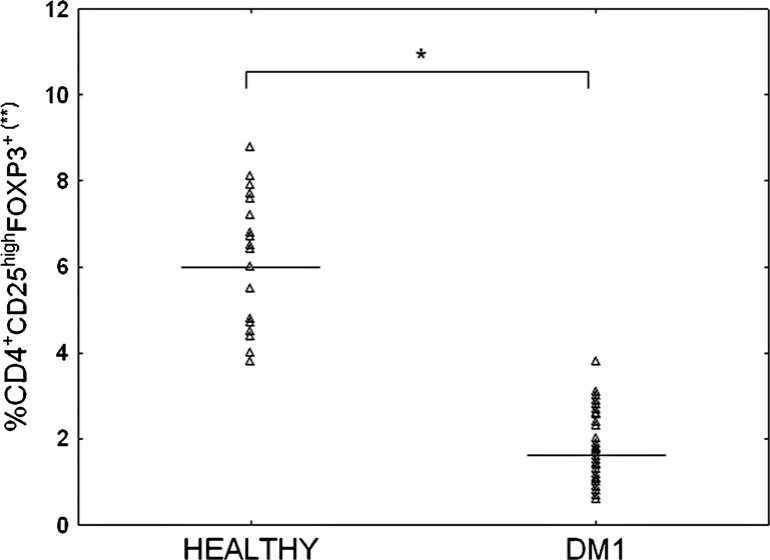
Frequency of CD4^+^CD25^high^FOXP3^+^ T cells in peripheral blood of diabetic type 1 patients and healthy individuals. Fresh, resting PBMCs from diabetic type 1 patients (DM1) and healthy individuals were stained with anti-CD4, anti-CD25, and anti-FOXP3 mAbs and then the frequency of CD4^+^CD25^high^FOXP3^+^ cells among CD4^+^ lymphocytes was determined using flow cytometry. The mean percentage of cells (25/75 percentiles) in DM1 and healthy group was 1.6 (1.1/2.3) and 6 (4.5/7.2), respectively. Data were calculated with Mann-Whitney U test. *Horizontal lines* represent the mean frequency of cells. **The percentage of cells among peripheral blood CD4^+^ lymphocytes. *Indicates significant difference versus healthy group (*p* = 0.0001).

Peripheral blood from the two groups of children was analyzed with regard to the frequency of CD4^+^CD25^high^ T cells expressing FOXP3 transcription factor. As shown in Figs. 2 and 3, the frequencies of circulating CD4^+^CD25^high^FOXP3^+^ Tregs were significantly lower in DM1 patients in comparison to their healthy counterparts from the control group.

Our study was extended by performing the correlation analysis between frequency of peripheral blood Tregs and serum level of CRP, IL-12, and IL-18 in DM1 group. The results of this analysis are presented in Table 3. A significant inverse association between the percentage of regulatory CD4^+^CD25^high^FOXP3^+^ Tregs and serum level of CRP, IL-12, and IL-18 (*R* = −0.69; *R* = −0.42; and *R* = −0.66, respectively) was observed.

**Table 3:** The Results of the Correlation Analysis between CD4^+^CD25^high^FOXP3^+^ Treg Frequencies and Level OF Crp, Il-12 and Il-18 in Serum of Dm1 Young Patients

	CRP	IL-12	IL-18
The percentage of CD4^+^CD25^high^FOXP3^+^ cells (%)*	*R* = [−0.69] *p* < 0.05	*R* = [−0.42] *p* < 0.05	*R* = [−0.66] *p* < 0.05

The Spearman test was used to calculate the strength of correlation

*The percentage of cells among peripheral blood lymphocytes

## DISCUSSION

Chronic inflammation in type 1 diabetes has been confirmed by the number of studies [38–41]. Several proinflammatory cytokines were shown to be elevated in serum of diabetic patients with a new onset of diabetes or a longstanding disease [38, 42, 43]. IL-12 and IL-18 are additional two cytokines that have been shown to exert strong proinflammatory activity and that synergize in action with each other, as well as with TNF-α or IL-1 [44]. In the present study, patients with type 1 diabetes had higher serum level of IL-12 and IL-18 in comparison to the healthy subjects from the control group. What’s more, serum levels of IL-12 and IL-18 were positively associated with CRP, which is one of the most important biomarkers of chronic inflammation [40, 42]. Recently, Devaraj *et al.* showed that CRP has the ability to polarize human monocytes towards proinflammatory M1 phenotype [45]. The overproduction of CRP may disrupt the balance between M1 and M2 macrophages and thus induce the production of IL-12 and/or IL-18 in an indirect way [10]. Our results demonstrating increased circulating IL-12 and IL-18 concentrations in DM1 patients are consistent with the studies done by Blazhev *et al.* [46], which showed that the levels of IL-12 and IL-18 have been increasing along with DM1 progression [46]. IL-12 as well as IL-18 may have a role in pathophysiology of the late microvascular complications; however, studies have yielded contradictory findings so far [24, 47, 48].

Besides CRP, a positive relationship between IL-12 serum level and glycosylated hemoglobin was found. However, we found no significant correlation between plasma IL-18 and HbA1c. HbA1c is an indicator of metabolic control and is measured to provide an index of average blood glucose for the previous 3 to 4 months. Esposito *et al.* have shown that acute hyperglycemia, contrary to chronic state, raised IL-18 level to a peak at 2 h, which returned to basal value after next hour [49]. This could explain the observed lack of correlation between concentration of IL-18 and HbA1c in our patient group. Similar findings regarding no association between serum IL-18 concentrations and the level of glycemic control were reported by other groups [23, 50, 51]. However, there are some studies with opposite results, suggesting that elevated IL-18 levels could, at least in part, contribute to the development of diabetic complications [47, 48]. This is even more likely as the value of HbA1c is strongly associated with complications of diabetes [52]. DM1 patients with poor glycemic control are characterized by elevated serum level of TNF-α, so more intense inflammatory response [24]. Importantly, IL-12, as well IL-18 were shown to induce the synthesis of TNF-α which makes them complications-accelerating indirect factors [48, 53]. In addition, IL-12 was found to be involved in the progression of retinopathy [8] and CRP may upregulate its synthesis [54], which is in agreement with our results.

In view of the facts that control of inflammatory response is closely related to regulatory T cells, we’ve decided to analyze the CD4^+^CD25^high^FOXP3^+^ T cell subset in the context of IL-12 and IL-18 cytokine milleu in DM1 patients. We found a decreased percentage of these cells in peripheral blood of diabetic type 1 patients in comparison to their healthy counterparts, but what’s more interesting we observed the inverse association between serum level of IL-12, IL-18, and frequencies of Tregs. It is difficult to decide whether Tregs are unable to suppress production of inflammatory cytokines, or IL-12/IL-18 has detrimental effect on Tregs numbers. There are only few published mouse model studies on the association between IL-12/IL-18 cytokines and regulatory T cell subset.

A possible link between IL-12 and the induction of Tregs was suggested by Morrow *et al.* [54], who showed that IL-12 increased the number of splenic regulatory T cells in immunized mice [54]. Zhao *et al.* [55] also demonstrated that IL-12 signaling pathway may have a role in regulation of Tregs numbers. The authors showed that mice lacking β2 chain of IL-12 receptor had more CD4^+^CD25^−^ effector T cells but fewer CD4^+^CD25^+^ Tregs than wild-type mice upon activation [55]. Previous studies by King *et al.* [56] suggested that IL-12 acts directly on CD4^+^CD25^−^ effector T cells rather than Tregs. IL-12 restores CD4^+^CD25^−^ T cell activation, even in the presence of regulatory T cells [56]. In other studies, IL-12 was shown to induce IFN-γ production by Tregs *in vitro* and *in vivo* [57–59]. Induction of IFN-γ expression by Tregs upon IL-12 treatment reduced Treg numbers and expression of FOXP3 transcription factor [57, 58]. Recent study by Zhao *et al.* [59] showed that IL-12 increased IL-2R expression on effector T cells, diminished its expression on Tregs, and decreased IL-2 production by effector T cells [59]. IL-2 is essential for the maintenance of regulatory T cells [30]. Low level of IL-2 may contribute to reduced Treg proliferation, which perhaps leads to their diminished numbers.

Similarly to IL-12, IL-18 was shown to increase the ratio of effector T cells to Tregs [60]. However, there are contradictory results showing that IL-18 is essential for inducing antigen-specific regulatory T cells and oral tolerance [61]. In contrast to these data, Zeiser *et al.* [62] demonstrated that IL-18 is not required for Treg expansion [62]. One of the know feature of IL-18 is its ability to stimulate the Th17 cells [10]. Th17 cells are involved in the pathogenesis of inflammatory and autoimmune diseases, and they also predominate in patients with type 1 diabetes, which was shown by us and others [63, 64]. Upregulated Th17 immune response may have impact on Treg numbers.

In view of the limited data regarding the relation between proinflammatory IL-12/IL-18 and regulatory T cells, further studies in humans are needed to properly verify this. However, the results of our analysis lead us to conclude that patients with type 1 diabetes have enhanced inflammatory response, which is manifested by increased values of CRP, HbA1c, IL-12, and IL-18. These mediators of inflammation may have direct or indirect impact on regulatory T cell subset, which may contribute to their reduced frequency in peripheral blood.

In the larger context, the data presented by us are mainly observation, and future *in vitro* studies are needed to determine the impact of IL-12 and IL-18 on Tregs quantitative as well as qualitative changes.

## References

[1] King GL (2008). The role of inflammatory cytokines in diabetes and its complications. Journal of Periodontology.

[2] Watford WT, Moriguchi M, Morinobu A, O’Shea JJ (2003). The biology of IL-12: Coordinating innate and adaptive immune responses. Cytokine & Growth Factor Reviews.

[3] Hölscher C (2004). The power of combinatorial immunology: IL-12 and IL-12-related dimeric cytokines in infectious diseases. Medical Microbiology and Immunology.

[4] Adorini L (2001). Interleukin 12 and autoimmune diabetes. Nature Genetics.

[5] Alleva DG, Pavlovich RP, Grant C, Kaser SB, Beller DI (2000). Aberrant macrophage cytokine production is a conserved feature among autoimmune-prone mouse strains: elevated interleukin (IL)-12 and an imbalance in tumor necrosis factor-alpha and IL-10 define a unique cytokine profile in macrophages from young nonobese diabetic mice. Diabetes.

[6] Wu HP, Chen CH, Hsieh HC, Liu YC (2008). Effects of insulin and glucose on cytokine production from peripheral blood mononuclear cells. Chang Gung Medical Journal.

[7] Wu HP, Kuo SF, Wu SY, Chuang DY (2010). High interleukin-12 production from stimulated peripheral blood mononuclear cells of type 2 diabetes patients. Cytokine.

[8] Gverović Antunica A, Karaman K, Znaor L, Sapunar A, Buško V, Puzović V (2012). IL-12 concentrations in the aqueous humor and serum of diabetic retinopathy patients. Graefe’s Archive for Clinical and Experimental Ophthalmology.

[9] Tsutsui H, Matsui K, Okamura H, Nakanishi K (2000). Pathophysiological roles of interleukin-18 for inflammatory liver diseases. Immunological Reviews.

[10] Boraschi D, Dinarello CA (2006). IL-18 in autoimmunity: Review. European Cytokine Network.

[11] Liu Z, Wang H, Xiao W, Wang C, Liu G, Hong T (2010). Thyrocyte interleukin-18 expression is up-regulated by interferon-γ and may contribute to thyroid destruction in Hashimoto’s thyroiditis. International Journal of Experimental Pathology.

[12] Zhang W, Cong XL, Qin YH, He ZW, He DY, Dai SM (2013). IL-18 upregulates the production of key regulators of osteoclastogenesis from fibroblast-like synoviocytes in rheumatoid arthritis. Inflammation.

[13] Wen D, Liu J, Du X, Dong JZ, Ma CS (2014). Association of interleukin-18 (-137G/C) polymorphism with rheumatoid arthritis and systemic lupus erythematosus: a meta-analysis. International Reviews of Immunology.

[14] Sawada M, Kawayama T, Imaoka H, Sakazaki Y, Oda H, Takenaka S, Kaku Y, Azuma K, Tajiri M, Edakuni N, Okamoto M, Kato S, Hoshino T (2013). IL-18 induces airway hyperresponsiveness and pulmonary inflammation via CD4+ T cell and IL-13. PLoS One.

[15] Kawayama T, Okamoto M, Imaoka H, Kato S, Young HA, Hoshino T (2012). Interleukin-18 in pulmonary inflammatory diseases. Journal of Interferon & Cytokine Research.

[16] Kanai T, Watanabe M, Okazawa A, Sato T, Hibi T (2001). Interleukin-18 and Crohn’s disease. Digestion.

[17] Kretowski A, Mironczuk K, Karpinska A, Bojaryn U, Kinalski M, Puchalski Z, Kinalska I (2002). Interleukin-18 promoter polymorphisms in type 1 diabetes. Diabetes.

[18] Ide A, Kawasaki E, Abiru N, Sun F, Kobayashi M, Fukushima T, Takahashi R, Kuwahara H, Kita A, Oshima K, Uotani S, Yamasaki H, Yamaguchi Y, Eguchi K (2004). Association between IL-18 gene promoter polymorphisms and CTLA-4 gene 49A/G polymorphism in Japanese patients with type 1 diabetes. Journal of Autoimmunity.

[19] Szeszko JS, Howson JM, Cooper JD, Walker NM, Twells RC, Stevens HE, Nutland SL, Todd JA (2006). Analysis of polymorphisms of the interleukin-18 gene in type 1 diabetes and Hardy–Weinberg equilibrium testing. Diabetes.

[20] Mojtahedi Z, Naeimi S, Farjadian S, Omrani GR, Ghaderi A (2006). Association of IL-18 promoter polymorphisms with predisposition to type 1 diabetes. Diabetic Medicine.

[21] Myhr CB, Hulme MA, Wasserfall CH, Hong PJ, Lakshmi PS, Schatz DA, Haller MJ, Brusko TM, Atkinson MA (2013). The autoimmune disease-associated SNP rs917997 of IL18RAP controls IFNγ production by PBMC. Journal of Autoimmunity.

[22] Esposito K, Nappo F, Giugliano F, Di Palo C, Ciotola M, Barbieri M, Paolisso G, Giugliano D (2003). Cytokine milieu tends toward inflammation in type 2 diabetes. Diabetes Care.

[23] Aso Y, Okumura K, Takebayashi K, Wakabayashi S, Inukai T (2003). Relationships of plasma interleukin-18 concentrations to hyperhomocysteinemia and carotid intimal-media wall thickness in patients with type 2 diabetes. Diabetes Care.

[24] Mahmoud RA, el-Ezz SA, Hegazy AS (2004). Increased serum levels of interleukin-18 in patients with diabetic nephropathy. The Italian Journal of Biochemistry.

[25] Moriwaki Y, Yamamoto T, Shibutani Y, Aoki E, Tsutsumi Z, Takahashi S, Okamura H, Koga M, Fukuchi M, Hada T (2003). Elevated levels of interleukin-18 and tumor necrosis factor-alpha in serum of patients with type 2 diabetes mellitus: Relationship with diabetic nephropathy. Metabolism.

[26] Fehérvari Z, Sakaguchi S (2004). CD4+ Tregs and immune control. Journal of Clinical Investigation.

[27] Prado C, de Paz B, López P, Gómez J, Rodríguez-Carrio J, Suáre A (2013). Relationship between FOXP3 positive populations and cytokine production in systemic lupus erythematosus. Cytokine.

[28] Sakaguchi S, Miyara M, Costantino CM, Hafler DA (2010). FOXP3+ regulatory T cells in the human immune system. Nature Reviews Immunology.

[29] Baecher-Allan C, Brown JA, Freeman CJ, Hafler DA (2001). CD4 + CD25high regulatory cells in human peripheral blood. Journal of Immunology.

[30] Miyara M, Gorochov G, Ehrenstein M, Musset L, Sakaguchi S, Amoura Z (2011). Human FoxP3+ regulatory T cells in systemic autoimmune diseases. Autoimmunity Reviews.

[31] Chavele KM, Ehrenstein MR (2011). Regulatory T-cells in systemic lupus erythematosus and rheumatoid arthritis. FEBS Letters.

[32] Huan J, Culbertson N, Spencer L, Bartholomew R, Burrows GG, Chou YK, Bourdette D, Ziegler SF, Offner H, Vandenbark AA (2005). Decreased FOXP3 levels in multiple sclerosis patients. Journal of Neuroscience Research.

[33] Ryba M, Marek N, Hak Ł, Rybarczyk-Kapturska K, Myśliwiec M, Trzonkowski P, Myśliwska J (2011). Anti-TNF rescue CD4 + Foxp3+ regulatory T cells in patients with type 1 diabetes from effects mediated by TNF. Cytokine.

[34] Eller K, Kirsch A, Wolf AM, Sopper S, Tagwerker A, Stanzl U, Wolf D, Patsch W, Rosenkranz AR, Eller P (2011). Potential role of regulatory T cells in reversing obesity-linked insulin resistance and diabetic nephropathy. Diabetes.

[35] Wang Y, Feng X, Bao S, Yi S, Kairaitis L, Tay YC, Rangan GK, Harris DC (2001). Depletion of CD4+ T cells aggravates glomerular and interstitial injury in murine adriamycin nephropathy. Kidney International.

[36] Wang YM, Zhang GY, Wang Y, Hu M, Wu H, Watson D, Hori S, Alexander IE, Harris DC, Alexander SI (2006). Foxp3-transduced polyclonal regulatory T cells protect against chronic renal injury from adriamycin. Journal of the American Society of Nephrology.

[37] Mahajan D, Wang Y, Qin X, Wang Y, Zheng G, Wang YM, Alexander SI, Harris DC (2006). CD4 + CD25+ regulatory T cells protect against injury in an innate murine model of chronic kidney disease. Journal of the American Society of Nephrology.

[38] Bierhaus A, Schiekofer S, Schwaninger M, Andrassy M, Humpert PM, Chen J, Hong M, Luther T, Henle T, Klöting I, Morcos M, Hofmann M, Tritschler H, Weigle B, Kasper M, Smith M, Perry G, Schmidt AM, Stern DM, Häring HU, Schleicher E, Nawroth PP (2001). Diabetes-associated sustained activation of the transcription factor nuclear factor-kappaB. Diabetes.

[39] Devaraj S, Glaser N, Griffen S, Wang-Polagruto J, Miguelino E, Jialal I (2006). Increased monocytic activity and biomarkers of inflammation in patients with type 1 diabetes. Diabetes.

[40] Jialal I, Devaraj S (2012). Circulating versus cellular biomarkers of inflammation in Type 1 diabetes: the superiority of C-reactive protein. Cytokine.

[41] Devaraj S, Cheung AT, Jialal I, Griffen SC, Nguyen D, Glaser N, Aoki T (2007). Evidence of increased inflammation and microcirculatory abnormalitie in patients with type 1 diabetes and their role in microvascular complications. Diabetes.

[42] Schramm MT, Chaturvedi N, Schalkwijk C, Giorgino F, Ebeling P, Fuller JH, Stehouwer CD (2003). The EURODIAB Prospective Complications Study Group: Vascular risk factors and markers of endothelial function as determinants of inflammatory markers in type 1 diabetes. Diabetes Care.

[43] Ishihara K, Hirano T (2002). IL-6 in autoimmune disease and chronic inflammatory proliferative disease. Cytokine & Growth Factor Reviews.

[44] Malaviya AM (2006). Cytokine network and its manipulation in rheumatoid arthritis. The Journal of the Association of Physicians of India.

[45] Devaraj S, Jialal I (2011). C-reactive protein polarizes human macrophages to an M1 phenotype and inhibits transformation to the M2 phenotype. Arteriosclerosis, Thrombosis, and Vascular Biology.

[46] Blazhev A, Nicoloff G, Petrova C, Jordanova-Laleva P (2006). Serum levels of interleukin 12 and interleukin 18 in diabetic Children. Diabetologia Croatica.

[47] Altinova AE, Yetkin I, Akbay E, Bukan N, Arslan M (2008). Serum IL-18 levels in patients with type 1 diabetes: Relations to metabolic control and microvascular complications. Cytokine.

[48] Katakami N, Kaneto H, Matsuhisa M, Yoshiuchi K, Kato K, Yamamoto K, Umayahara Y, Kosugi K, Hori M, Yamasaki Y (2007). Serum interleukin-18 levels are increased and closely associated with various soluble adhesion molecule levels in type 1 diabetic patients. Diabetes Care.

[49] Esposito K, Nappo F, Marfella R, Giugliano G, Giugliano F, Ciotola M, Quagliaro L, Ceriello A, Giugliano D (2002). Inflammatory cytokine concentrations are acutely increased by hyperglycemia in humans: Role of oxidative stress. Circulation.

[50] Kretowski A, Kinalska I (2003). Serum levels of interleukin-18–a potential marker of cardiovascular death–could be determined by genetic predisposition. Circulation.

[51] Dong G, Liang L, Fu J, Zou C (2007). Serum interleukin-18 levels are raised in diabetic ketoacidosis in Chinese children with type 1 diabetes mellitus. Indian Pediatrics.

[52] Renard E (2005). Monitoring glycemic control: the importance of self-monitoring of blood glucose. American Journal of Medicine.

[53] Jana M, Dasgupta S, Saha RN, Liu X, Pahan K (2003). Induction of tumor necrosis factor-alpha (TNF-alpha) by interleukin-12 p40 monomer and homodimer in microglia and macrophages. Journal of Neurochemistry.

[54] Morrow MP, Pankhong P, Laddy DJ, Schoenly KA, Yan J, Cisper N, Weiner DB (2009). Comparative ability of IL-12 and IL-28B to regulate Treg populations and enhance adaptive cellular immunity. Blood.

[55] Zhao Z, Yu S, Fitzgerald DC, Elbehi M, Ciric B, Rostami AM, Zhang GX (2008). IL-12R beta 2 promotes the development of CD4 + CD25+ regulatory T cells. Journal of Immunology.

[56] King IL, Segal BM (2005). Cutting edge: IL-12 induces CD4 + CD25- T cell activation in the presence of T regulatory cells. Journal of Immunology.

[57] Oldenhove G, Bouladoux N, Wohlfert EA, Hall JA, Chou D, Dos Santos L, O’Brien S, Blank R, Lamb E, Natarajan S, Kastenmayer R, Hunter C, Grigg ME, Belkaid Y (2009). Decrease of Foxp3+ Treg cell number and acquisition of effector cell phenotype during lethal infection. Immunity.

[58] Feng T, Cao AT, Weaver CT, Elson CO, Cong Y (2011). Interleukin-12 converts Foxp3+ regulatory T cells to interferon-gamma-producing Foxp3+ T cells that inhibit colitis. Gastroenterology.

[59] Zhao J, Zhao J, Perlman S (2012). Differential effects of IL-12 on Tregs and non-Treg T cells: roles of IFN-γ, IL-2 and IL-2R. PLoS One.

[60] Carroll RG, Carpenito C, Shan X, Danet-Desnoyers G, Liu R, Jiang S, Albelda SM, Golovina T, Coukos G, Riley JL, Jonak ZL, June CH (2008). Distinct effects of IL-18 on the engraftment and function of human effector CD8 T cells and regulatory T cells. PLoS One.

[61] Tsuji NM, Nowak B (2004). IL-18 and antigen-specific CD4(+) regulatory T cells in Peyer’s patches. Annals of the New York Academy of Sciences.

[62] Zeiser R, Zambricki EA, Leveson-Gower D, Kambham N, Beilhack A, Negrin RS (2007). Host-derived interleukin-18 differentially impacts regulatory and conventional T cell expansion during acute graft-versus-host disease. Biology of Blood and Marrow Transplantation.

[63] Ryba-Stanisławowska M, Skrzypkowska M, Myśliwiec M, Myśliwska J (2013). Loss of the balance between CD4(+)Foxp3(+) regulatory T cells and CD4(+)IL17A(+) Th17 cells in patients with type 1 diabetes. Human Immunology.

[64] Honkanen J, Nieminen JK, Gao R, Luopajarvi K, Salo HM, Ilonen J, Knip M, Otonkoski T, Vaarala O (2010). IL-17 immunity in human type 1 diabetes. Journal of Immunology.

